# Detection of somatic variants and *EGFR* mutations in cell-free DNA from non-small cell lung cancer patients by ultra-deep sequencing using the ion ampliseq cancer hotspot panel and droplet digital polymerase chain reaction

**DOI:** 10.18632/oncotarget.22456

**Published:** 2017-11-15

**Authors:** Jae Sook Sung, Hyon Yong Chong, Nak-Jung Kwon, Hae Mi Kim, Jong Won Lee, Boyeon Kim, Saet Byeol Lee, Chang Won Park, Jung Yoon Choi, Won Jin Chang, Yoon Ji Choi, Sung Yong Lee, Eun Joo Kang, Kyong Hwa Park, Yeul Hong Kim

**Affiliations:** ^1^ Cancer Research Institute, Korea University, Seoul, Republic of Korea; ^2^ Macrogen, Seoul, Republic of Korea; ^3^ Division of Oncology/Hematology, Department of Internal Medicine, Korea University Anam Hospital, Korea University College of Medicine, Seoul, Republic of Korea; ^4^ Department of Internal Medicine, Guro Hospital, Korea University, Seoul, Republic of Korea

**Keywords:** non-small-cell lung carcinoma, multiplex polymerase chain reaction, missense mutation, cell-free DNA, molecular targeted therapy

## Abstract

Highly sensitive genotyping assays can detect mutations in cell-free DNA (cfDNA) from cancer patients, reflecting the biology of each patient’s cancer. Because circulating tumor DNA comprises a small, variable fraction of DNA circulating in the blood, sensitive parallel multiplexing tests are required to determine mutation profiles. We prospectively examined the clinical utility of ultra-deep sequencing analysis of cfDNA from 126 non-small cell lung cancer (NSCLC) patients using the Ion AmpliSeq Cancer Hotspot Panel v2 (ICP) and validated these findings with droplet digital polymerase chain reaction (ddPCR). ICP results were compared with tumor tissue genotyping (TTG) results and clinical outcomes. A total of 853 variants were detected, with a median of four variants per patient. Overall concordance of ICP and TTG analyses was 90% for EGFR exon 19 deletion and 88% for the L858R mutation. Of 34 patients with a well-defined *EGFR* activating mutation defined based on the results of ICP and TTG, 31 (81.6%) showed long-term disease control with EGFR TKI treatment. Of 56 patients treated with an EGFR tyrosine kinase inhibitor (TKI), the presence of the *de novo* T790M mutation was confirmed in 28 (50%). Presence of this *de novo* mutation did not have a negative effect on EGFR TKI treatment. Ultra-deep sequencing analysis of cfDNA using ICP combined with confirmatory ddPCR was effective at defining driver genetic changes in NSCLC patients. Comprehensive analysis of tumor DNA and cfDNA can increase the specificity of molecular diagnosis, which could translate into tailored treatment.

## INTRODUCTION

Lung cancer is the most common cause of global cancer-related mortality and resulted in 17,177 deaths in Korea in 2013 [1]. More than 85% of lung cancer cases are currently classified as non-small-cell lung cancer (NSCLC), for which the predicted 5-year survival rate is 15.9% [2]. NSCLC is characterized by a unique pattern of genetic driver mutations, some of which are used to predict prognosis or for targeted treatment [3–5]. In lung adenocarcinoma, multiple genetic alterations have already been identified as therapeutic targets, including mutations of the *EGFR* gene and rearrangement of the *ALK* and *ROS1* genes [6–8]. In addition, several other target oncogenes with potential prognostic roles in lung adenocarcinoma, including *MET*, *PIK3CA*, and *RET*, have also been described, and target agents are currently under development [9]. Given the increased availability of various targeted agents, comprehensive characterization of mutations in clinically actionable genes and key cancer pathways can be helpful for prognosis prediction and selection of the appropriate treatment agents [10].

Next-generation sequencing (NGS)-based platforms allow parallel multigene testing for the molecular diagnosis of cancer [11]. Compared with conventional gene-specific assays, NGS platforms are more sensitive, have a lower per sample cost, and allow a broader range of mutations to be detected [12]. In particular, targeted NGS platforms are cost-effective and allow rapid simultaneous detection of multiple mutations in various genes with high reproducibility and sensitivity [12].

Cell-free DNA (cfDNA) present in the blood stream shows great potential as a useful cancer marker for molecular diagnosis and cancer progression monitoring [13–16]. Even small tumors containing as few as 50 million cells release sufficient DNA to be detected in the blood, whereas tumors of this size fall well below the detection limits of standard radiological techniques [14]. Several studies have demonstrated that mutations detected in cfDNA, including *EGFR* mutations, are highly concordant with those detected in lung cancer tissues [17–19], indicating that cfDNA as a liquid biopsy is a feasible and minimally invasive alternative to tissue biopsy. Concordance rates for various gene mutations in cfDNA and tumor DNA ranged from 64% to 98% according to the type of platform and genes [20–22]. Blood-based genotyping is a technology ready for use in clinical decision-making in patients with NSCLC, especially droplet digital polymerase chain reaction (ddPCR)-based assays [23]. Although, sensitive blood-based ddPCR assays can be useful for monitoring treatment response or early development of resistance in a noninvasive way [24], these assays have limitations in multiplex gene testing. In contrast, analyzing cfDNA with NGS technology allows concurrent high throughput examination of various genes at a low cost [25–27]. Because the lowest mutant allele frequency in cfDNA for deletion of exon 19 of *EGFR* and the L858R mutation is 0.005% and 0.003%, respectively [24], targeted NGS requires ultra-deep sequencing (> 20,000x coverage) to detect these very low frequency mutations. In this case, ddPCR could be utilized as a validation test to overcome possible sequencing errors or borderline significant results in targeted NGS ultra-deep sequencing.

In this study, we prospectively examined the clinical utility of ultra-deep sequencing analysis of cfDNA from 126 NSCLC patients using Ion AmpliSeq Cancer Hotspot Panel v2 (ICP; Ion Torrent) and the Proton platform; this panel covers 2,800 COSMIC mutations from 50 cancer genes. ICP results were validated with ddPCR.

## RESULTS

### Patient characteristics

Ultra-deep targeted sequencing of cfDNA from 126 NSCLC patients (Table 1) was performed using ICP. The average age of the patients was 63.98 years, with a standard deviation of 11.12. Of the patients, 65.9% were male, 59.5% were smokers, 76% had adenocarcinomas, and 85.7% had stage IV cancer. Fifty-six patients (44.5%) underwent EGFR tyrosine kinase inhibitor (TKI) treatment based on the treatment guidelines of the Korean Health Insurance Review & Assessment. Twenty-five patients received EGFR TKI treatment as a first line treatment, while others (31/56) were treated with EGFR TKIs as second line treatment or more. TTG results for the *EGFR* gene obtained using FFPE were available from 100 patients. Of these patients, 34 had *EGFR* activating mutations (exon 19 deletion or L858R). cfDNA extracted from three patients failed to pass DNA QC for ICP analysis. The average yield of cfDNA in 500 µl serum was 58.44 ng (range, 8.23–282.80 ng).

**Table 1:** Clinical features of the 126 lung cancer patients

Characteristics		Number of Patients (%)
Age		
	Average	63.98 ± 11.12
Gender		
	Male	83 (65.87)
	Female	43 (34.13)
Smoking status		
	Smoker	75 (59.52)
	Never-smoker	51 (40.48)
Cell type		
	Adenocarcinoma	96 (76.19)
	Squamous cell carcinoma	20 (15.87)
	Others	10 (7.94)
Stage		
	I-III	13 (10.32)
	IV	108 (85.71)
	Relapse	5 (3.97)
EGFR TKI treatment		
	First line	25 (19.84)
	Second or beyond	31 (24.60)
	Not treated (without EGFR TKI treatment)	70 (55.56)
TTG results for *EGFR* activating mutations		
	Wild type	66 (52.38)
	Mutant type	34 (26.98)
	Not done	26 (20.63)

TTG: tumor tissue genotyping.

### Ion ampliSeq cancer hotspot panel analysis of cfDNA with the ion torrent proton system

In the 123 cfDNA samples analyzed in this study, the distribution of sequence lengths was between 60 and 170 bp (Supplementary Figure 1A). The GC content across all bases was roughly 30% (Supplementary Figure 1B), and quality scores across all bases are shown in Supplementary Figure 1C. Targeted sequencing using the ICP panel generated approximately 604 Mb per sample with an average of 92.27% on target. Sequences of all samples achieved a mean depth of 22,868x. We determined all mutations in four buffy coat samples of germline mutations. Furthermore, we used a cfDNA reference standard set with the following specific mutations: *EGFR* exon 19 deletion, L858R, T790M, *KRAS* G12D, *NRAS* Q61K, *PIK3CA* E545K (5%, 1%, and 0.1%) or wild type (0%) and determined the accuracy of ICP and ddPCR (Supplementary Table 1). Both platforms detected mutations at the 5% and 1% level with fairly good accuracy. Ultra-sensitive ICP analysis of a wild type sample with *EGFR* T790M, L858R, *KRAS* G12D, *NRAS* Q61K, and *PIK3CA* E545K primers showed false positive findings with a very low frequency, mostly < 0.05%. Thus, low-frequency ICP data (< 0.1%) require validation using other specific platforms, such as ddPCR.

### Detection of somatic mutations from cfDNA in 123 non-small cell lung cancer patients

**Figure 1:**
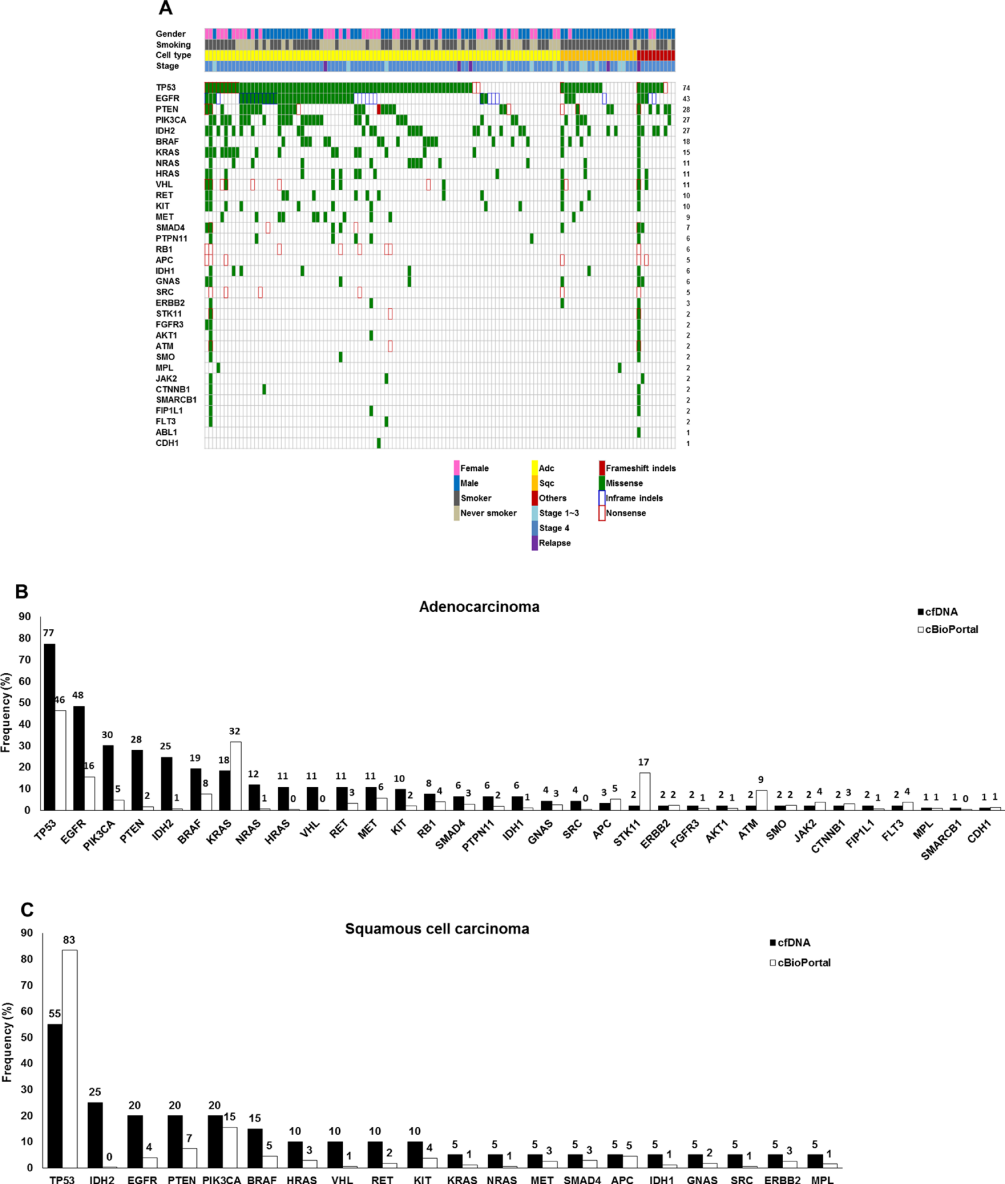
Genetic variations in the cfDNA of 123 patients with NSCLC based on ICP analysis (**A**) Summary of genetic variations in the 123 NSCLC patients. (**B**) Frequencies of variations in 93 lung adenocarcinomas. (**C**) Frequencies of variations in 20 squamous cell carcinomas.

According to the ICP results, 12 patients had no somatic variants in any of the 50 genes evaluated. A total of 853 variants were detected, with a median of four variants per patient. Variants were detected in 34 genes, with *EGFR* mutations prevalent in 12% of total variants. As shown in Figure 1A, variants were mainly identified in *TP53* (74%), *EGFR* (43%), *PTEN* (28%), *PIK3CA* (27%), *IDH2* (27%), *BRAF* (18%), *KRAS* (15%), *NRAS* (11%), *HRAS* (11%), *VHL* (11%), *KIT* (10%), and *RET* (10%) with the cut-off criteria of variant frequency > 0.1% and *p* < 0.01. Most variants were missense mutations. In the adenocarcinoma group, variants in various genes including *TP53* (77%), *EGFR* (48%), *PTEN* (28%), *PIK3CA* (30%), *IDH2* (25%), *BRAF* (19%), *KRAS* (18%), *NRAS* (12%), *HRAS* (11%), *VHL* (11%), *RET* (11%), *MET* (11%), and *KIT* (10%) were identified (Figure 1B). *PIK3CA* mutations were detected at a six-fold higher frequency in cfDNA than lung adenocarcinoma tissues based on data in the cBioPortal for Cancer Genomics database [28]. Similar patterns of genetic variants were detected in 20 squamous cell carcinoma patients (*TP53* (55%), *EGFR* (20%), *PTEN* (20%), *PIK3CA* (20%), *IDH2* (25%), *BRAF* (15%), *HRAS* (10%), *VHL* (10%), *RET* (10%), and *KIT* (10%)) (Figure 1C). Detailed information about the variants is provided in Supplementary Table 2A. In four squamous cell carcinoma patients with *EGFR* mutations, two had the T790M mutation without *EGFR* activating mutations, while the other two were positive for exon 19 deletion and L858R, respectively. Median numbers of mutations were four in adenocarcinoma patients, two in squamous cell carcinoma patients, and three in patients with other lung cancer types (Supplementary Table 2B). There were two patients who had more than 100 genetic variants in their cfDNA. Patient #071, who was diagnosed with lung adenocarcinoma, stage IV due to metastases to the brain, adrenal gland, and bone (multiple spines, pelvic bone, humerus, femur, and ribs), had 125 variants including *EGFR* mutations (L858R and T790M) and multiple *KRAS* mutations. One hundred one variants were detected in patient #083, who was diagnosed with adenosquamous cell carcinoma, stage IB (T2aN0M0). The 4.7-cm tumor was resected, and blood was obtained before surgical resection.

### Detection of *EGFR* mutations by cfDNA ICP analysis and comparison with TTG results

*EGFR* activating mutations detected by cfDNA ICP analysis and TTG data from 123 patients are presented in Supplementary Table 3. *EGFR* activating mutations were not detected in either analysis in 47 patients. Fourteen patients showed *EGFR* mutations only in TTG, while 19 patients showed *EGFR* mutations only in cfDNA. Seventeen patients had activating mutations in both analyses. Exon 19 deletion was found in 24 patients with a median variant frequency of 1.19% (range, 0.18%–44.82%), and the L858R mutation was identified in 30 patients with a median variant frequency of 0.34% (range, 0.16%–28.49%) by ICP analysis. Interestingly, *EGFR* exon 19 deletion and the L858R mutation were simultaneously identified in seven patients (#006, #076, #087, #101, #116, #119, and #123) using ICP analysis. In three patients (#051, #117, and #120), the type of activating mutation was different between the two tests. *EGFR* T790M mutation was detected in 30 patients (24.4%) based on ICP analysis of cfDNA, but not in TTG analysis of tumor tissue. In 23 patients, T790M mutations and *EGFR* activating mutations were present, while in seven patients, no *EGFR* activating mutations were present. Sensitivity of *EGFR* exon 19 using ICP was 72.73%, and that of the L858R mutation was 53.57%. Specificity of *EGFR* exon 19 deletion and L858R mutation using ICP was 93.94% and 98.91%, respectively. Overall concordance between ICP and TTG analyses was 90.08% for *EGFR* exon 19 deletion and 88.33% for the L858R mutation. Of 23 patients without TTG results, eight had *EGFR* activating mutations based on cfDNA ICP analysis.

### Validation with ddPCR and clinical significance of *EGFR* mutations detected in cfDNA

In cases with discordant *EGFR* activation mutations based on ICP and TTG analyses, we performed ddPCR to validate the results. First, to validate negative results for *EGFR* activation mutations and variants with a low frequency in ICP in patients with *EGFR* activating mutations according to TTG, ddPCR was carried out using cfDNA samples (Table 2). Among 14 patients who were positive for *EGFR* activating mutations according to TTG but negative according to ICP, 10 had a very low frequency (< 0.05%) or a *p* value > 0.01 for the same type of activating *EGFR* mutation in ICP as that detected by TTG. Among these 10 patients, *EGFR* mutations in cfDNA were confirmed in five (minimum frequency 0.01%) by ddPCR. In the other four patients with *EGFR* activating mutations according to TTG but low-frequency activating *EGFR* mutations in ICP, ddPCR did not detect any mutation of the *EGFR* gene. ddPCR failed in one patient (#009). Among four patients who were positive for exon 19 deletion according to TTG but no detectable exon 19 deletion according to ICP, one (#112) showed s 0.08% frequency of exon 19 deletion in ddPCR using a cfDNA sample. However, L858R mutation was detected in the other three patients (#057, #098, and #107) by ddPCR using a cfDNA sample, which was a different type of *EGFR* activating mutation from that of TTG.

**Table 2:** Results of validation by ddPCR in patients with discordant EGFR mutation status between TTG and ICP and those with low-frequency mutations based on ICP

	EGFR activating mutations status								
Patient No.	Exon 19 deletion			L858R			T790M		
	TTG	ICP (%)	ddPCR (%)	TTG	ICP (%)	ddPCR (%)	TTG	ICP (%)	ddPCR (%)
#004	Mut	W (0.004)	0.040	W	W (0.016)	0.010	W	W (0.037)	0
#048	Mut	W (0.050)	0.020	W	W (0.013)	0.020	W	W (0.039)	0.040
#093	Mut	W (0.044)	0.124	W	W (0.035)	ND	W	W (0.150)	ND
#121	Mut	W (0.120)	39.000	W	W (0.004)	0	W	W (0.060)	0
#032	W	W (0.079)	ND	Mut	W (0.055)	0.009	W	W (0.025)	ND
#009	Mut	W (0.009)	Failed	W	W (0)	ND	W	W (0.059)	ND
#040	W	W (0.029)	ND	Mut	W (0.024)	0	W	W (0.014)	ND
#061	Mut	W (0.018)	0	W	W (0.023)	ND	W	W (0.022)	ND
#113	Mut	W (0.048)	0	W	W (0.024)	0	W	W (0.037)	0.030
#111	W	W (0.059)	ND	Mut	W (0.005)	0	W	W (0.053)	0.060
#112	Mut	W (0)	0.080	W	W (0.005)	0	W	W (0.053)	NA
#057	Mut	W (0)	0	W	W (0.023)	0.005	W	W (0.049)	0.039
#098	Mut	W (0)	0	W	W (0.007)	0.008	W	W (0.066)	0.021
#107	Mut	W (0)	0	W	W (0.028)	0.032	W	W (0.048)	0.100
#087	Mut	Mut (44.819)	58.000	W	Mut (3.055)	0.024	W	Mut (1.480)	0
#119	Mut	Mut (3.341)	0.800	W	Mut (0.196)	0.120	W	W (0.044)	NA
#006	W	Mut (0.247)	0.034	W	Mut (0.226)	0	W	W (0)	ND
#076	Mut	Mut (0.180)	0.800	W	Mut (0.162)	0	W	Mut (0.417)	0.230
#101	W	Mut (2.068)	0	Mut	Mut (2.339)	1.690	W	W (0)	ND
#116	W	Mut (3.172)	0.500	Mut	Mut (2.315)	Failed	W	W (0)	ND
#123	W	Mut (0.539)	0	Mut	Mut (0.206)	Failed	W	W (0.023)	NA

ND: Not done; NA: Not available.

Confirmatory ddPCR analysis was also performed in seven patients with both types of *EGFR* activating mutations based on ICP. Only exon 19 deletions were detected in patients #87 and #119 by TTG. However, these patients were positive for exon 19 deletion and L858R in ICP analysis; this was confirmed by ddPCR. Patient #116 was diagnosed with the L858R mutation only in TTG, but both activating mutations were positive with a variant frequency of exon 19 deletion of 3.17% by ICP and 0.50% by ddPCR. This patient was positive for the L858R mutation by ICP with a variant frequency of 2.32%, but the ddPCR assay failed. These three patients appeared to harbor both types of activating mutations simultaneously in metastatic site cancer cells and the primary tumor. Additionally, two patients (#004 and #048) had both types of activating mutations based on ddPCR (Table 2). Three patients (#006, #076, and #101) with both *EGFR* activating mutations in ICP analysis were positive for one type of *EGFR* activating mutation in ddPCR using the same cfDNA, indicating that the ICP results were false positive in one of the three patients. Confirmatory ddPCR failed in patient #123.

### Validation of discrepancy in tumor tissue and serum *EGFR* mutation status

In five patients with detectable activating *EGFR* mutations based on TTG, different types of activating mutations were found in ICP analysis using cfDNA (patients #051, #107, #117, #119, and #120). However, no activating *EGFR* mutations were detected in ddPCR using cfDNA from two of these patients ( #051 and #120), while ddPCR failed in patient #117. To confirm the discrepancy between tumor tissue and serum *EGFR* mutation status in those patients, separate ICP and ddPCR analyses using tumor tissue DNA were carried out in four patients with available remaining tumor tissue (Table 3). ICP analysis using tumor tissue DNA from patient #120 revealed the presence of the L858R mutation, the same mutation found by TTG, with a variant frequency of 2.26%. ddPCR confirmed the presence of the L858R mutation in patient #120 with a variant frequency of 2.13% in tumor tissue DNA. In patient #120, blood-based ultra-deep sequencing and ddPCR failed to demonstrate a circulating L858R mutation, which was present in the primary tumor tissue at low frequency. Exon 19 deletion was not detected in patient #051 by ICP, while exon 19 deletion mutation was barely detected in patient #051 with a variant frequency of 0.03% in tumor tissue DNA. Considering the detection limits of conventional PCR assay using tumor tissue DNA, the TTG result of patient #051 might be a false positive result. Patients #119 and #107 harbored both activating *EGFR* mutations and the T790M mutation in tissue. However, the major clone of *EGFR* mutation was found with other minor clones in cfDNA from patient #119, and only minor mutation clones were noted in cfDNA from patient #107. Comparison of the mutation status of *EGFR* from tissue and serum samples in these four patients using both platforms revealed that results were reproducible and well matched between platforms. Based on these results, it is suggested that DNA from the primary tumor might not be released into the blood in some cases. More importantly, mutations detected by ultra-deep ICP analysis using cfDNA with a variant frequency less than 1% need to be validated by ddPCR.

**Table 3:** Validation of EGFR mutations using ICP and ddPCR analyses of tissue and cfDNA

Patient No.	EGFR	Tissue			cfDNA	
		TTG	ICP	ddPCR	ICP	ddPCR
#051	E19 deletion	Mut	W	0.03%	W	W
	T790M	W	0.11%	0.17%	0.44%	0.05%
	L858R	W	W	W	0.21%	W
#107	E19 deletion	Mut	24.32%	24.60%	W	W
	T790M	W	0.03%	0.03%	0.05%	0.10%
	L858R	W	0.02%	W	0.03%	0.03%
#119	E19 deletion	Mut	81.01%	77.20%	3.34%	0.80%
	T790M	W	0.33%	0.05%	0.04%	NA
	L858R	W	0.03%	W	0.20%	0.12%
#120	E19 deletion	W	W	W	0.39%	W
	T790M	W	0.15%	0.31%	0.19%	W
	L858R	Mut	2.26%	2.13%	0.01%	W

NA: Not available.

### Treatment outcome after EGFR TKI treatment according to comprehensive *EGFR* activating mutation status

We determined the *EGFR* activating mutation status of 56 patients treated with an EGFR TKI based on the TTG and ICP results. We also performed ddPCR using samples from these 56 patients to confirm the results (Supplementary Table 4). Of 38 patients with *EGFR* activating mutations, 31 (81.6%) showed a partial response or stable disease, and 11 are still undergoing EGFR TKI treatment. Of 18 patients with the wild-type *EGFR* gene, five (27.8%) showed a partial response or stable disease with EGFR TKI treatment, and two are still being treated. In contrast, of 29 patients with an *EGFR* activating mutation based on TTG that were treated with an EGFR TKI, 22 (75.9%) showed partial response (PR) or stable disease (SD). The *de novo* mutation T790M was detected in 28 patients (50%) by ddPCR. Twenty patients who had lower variant frequency than the cut-off level of the T790M mutation in ICP were confirmed by ddPCR to be positive for this mutation. Of the 14 patients positive for the T790M mutation based on ICP analysis, eight were confirmed to have this mutation by ddPCR. In the 28 T790M mutation-positive patients, 16 (57.1%) showed PR or SD to EGFR TKI treatment.

## DISCUSSION

In this prospective study, we explored the possibility of using targeted ultra-deep sequencing to identify driver genetic changes in the serum of NSCLC patients and validated these results with ddPCR. Recently, several groups have assessed genomic variations in lung cancer patients by NGS of cfDNA [20, 29, 30]. However, these studies only evaluated a small number of patients, and sequencing depth was only 10,000x. To our knowledge, this study is the first to prospectively assess the possibility of detecting cfDNA genetic variants by ultra-deep sequencing (mean depth 22,868x) in NSCLC patients. Furthermore, we validated the results by ddPCR assay and correlated the presence of specific mutations with clinical outcome.

Although many studies have evaluated the concordance between cfDNA and tumor tissue DNA mutations, the results from tumor tissue DNA should not be used as a reference to judge the sensitivity or specificity of an assay used for cfDNA analysis. Discordance in the detection of *EGFR* and *KRAS* mutations between the primary tumor and corresponding metastases has been shown to be as high as 28% and 24% in 25 patients with metastatic NSCLC, respectively [31]. Hence, it is more important to use complementary mutation profiles acquired from tumor tissue and blood-based genomic sources to make clinical decisions. Schwaederie and colleagues reported that tumor- and blood-based analyses could independently detect alterations not found in the other test, stressing the clinical value and complementary nature of the techniques [32]. Moreover, prospective evaluation to determine the optimal depth at which to demonstrate clinical significance with confirmatory validation is needed. Finally, those approaches should be validated by examining clinical outcome.

Several studies have proposed that highly sensitive genotyping assays can detect mutations in cfDNA from cancer patients, possibly reflecting the biology of each patient’s cancer [14, 33–35]. Because circulating tumor DNA comprises a small, variable fraction of total DNA circulating in the blood, and mutant DNA molecules account for 0.02% to 0.1% of all DNA assayed [30, 36], sensitive methods are necessary to identify the mutations in this small fraction [30]. Although highly sensitive test platforms such as ddPCR have proven clinical utility with a rapid turn-around time and reliability, parallel multiplexing testing is also required to determine the mutation profile of each patient. Using an NGS platform, increasing sequencing depth can increase the sensitivity for detecting low-frequency mutations. However, a challenge faced by highly sensitive genotyping assays is the detection of low-prevalence mutant alleles of unknown clinical significance. Furthermore, deep-sequencing can result in a high rate of erroneous base calls. The challenge of false-positive results is even greater when analyzing blood-based cfDNA; because cfDNA is mostly of germline origin from ruptured benign cells, tumor-derived mutations are innately present at a low prevalence, lowering the signal-to-noise ratio of assays [19].

Using ultra-deep ICP analysis of cfDNA, we successfully detected driver genetic changes in NSCLC patients. The median number of mutations per patient was four. Interestingly, two patients had 125 and 101 variants, respectively. These patients had the *EGFR* L858R mutation and other *EGFR* mutations (T790M and/or D761Y) as well as multiple *KRAS* mutations.

In the cBioPortal for Cancer Genomics database, which includes data from eight NSCLC studies, variants of *TP53* (62%), *KRAS* (21%), *EGFR* (11%), *PIK3CA* (8%), *BRAF* (6%), and *PTEN* (5%) were reported to be major genetic changes [28]. In our study, *TP53* mutations were more commonly detected in adenocarcinoma (77%) than squamous cell carcinoma (55%) based on ultra-deep ICP analysis of cfDNA. The *TP53* mutation R273H was the most frequently detected mutation (36.6%; 34/93) in adenocarcinoma patients. *TP53* mutations M237V and Y234C were also commonly detected at the same rate–31.2% (29/93)–in adenocarcinoma patients. The frequency of *PIK3CA, NRAS, HRAS*, and *PTEN* mutations was higher in cfDNA than in lung cancer tissue in the cBioPortal database [28]. This result is consistent with that reported by Chen *et al.* [30]. While *IDH2* R140Q and *MET* Y1248C mutations were not present in the cBioPortal database, we found those mutations at frequencies of 27% and 9%, respectively, based on cfDNA analysis. Thus, *PIK3CA, NRAS, HRAS, PTEN*, *MET*, and *IDH2* R140Q mutations might be related to the metastasis process rather than primary tumor development. The most common mutations of *KRAS* in the cBioPortal database of tumor tissue are G12C and G12A [28]. In comparison, however, common mutations in *KRAS* in cfDNA analysis from Korean patients were G12S, G12C, and G13G. While two variants, *NRAS* Q61L and *HRAS* G13R, are present in the cBioPortal data, we detected G12D (10/123), G13D (5/123), and G60E (1/123) mutations in *NRAS* and G12S (13/123), G12D (4/123), G13D (3/123), and G13C (2/123) in *HRAS*. The mutational profile of tumor tissue based on cBioPortal data and that of cfDNA are different, possibly due to differences in genetic sources, as well as the ethnicity and stage of patients.

To validate these results, ddPCR was carried using the corresponding cfDNA samples. Using this approach, we were able to define true positives and false positives based on concordance between the two tests. This approach could be used to confirm the mutation status in patients who do not have tissue available and in patients who only have wild-type driver mutations based on genetic analysis of tumor tissue. This is consistent with the high disease control rate of 81.6% found in our study based on comprehensive *EGFR* mutational profiling. In patients with wild-type *EGFR* based on comprehensive genetic analysis, TKI treatment stabilized disease in only 27.8%, and most patients showed progressive disease. Disease control rate of 81.6% was higher than that found in the group with *EGFR* activating mutations in primary tumor tissues based on TTG. Two different types of activating *EGFR* mutations (exon 19 deletion and L858R mutation) were simultaneously noted in five patients, which was confirmed by ddPCR. These findings show that tumor tissue DNA and cfDNA are heterogeneous, and that analysis of these two genetic sources can be complementary.

The *de novo* mutation T790M was detected in the cfDNA of 50% of patients who were treated with an EGFR TKI. Because these patients were not treated before blood was collected, this mutation did not develop in response to EGFR TKI treatment. This *de novo* T790M mutation did not have a negative effect on EGFR TKI treatment outcome.

In our study, we demonstrated that ultra-deep sequencing using ICP with a Proton system is a very sensitive method to identify somatic variants in cfDNA in NSCLC patients. Combined with confirmatory ddPCR, ultra-deep sequencing analysis of cfDNA using ICP could translate to a precision approach to determine the optimal treatment and predict prognosis.

## MATERIALS AND METHODS

### Patients and blood collection

Between September 2006 and July 2015, blood samples were prospectively collected from 126 NSCLC patients who provided informed consent to participate in this study (Table 1). The study was approved by the Institutional Review Boards of Korea University Anam Hospital and Guro Hospital. All samples and medical data used in this study were irreversibly anonymized. We attempted to minimize the time between collection of tissues and blood for genotyping in 100 patients who underwent tumor tissue genotyping (TTG) tests. However, five patients relapsed after resection of primary NSCLC, and resected tumors were utilized for TTG. Serum was separated within 2 hours from sample collection and stored at −80°C until use.

### *EGFR* mutation testing in tumor tissue DNA

TTG of the *EGFR* gene was performed in clinical laboratories of Korea University Anam Hospital and Guro Hospital. *EGFR* mutations in tumor tissue DNA were detected by direct sequencing (59 tests), PNA Clamp PCR (30 tests), or pyrosequencing (11 tests) of formalin-fixed paraffin-embedded tissue samples from 100 NSCLC patients. Twenty-five patients did not have available tumor tissue for *EGFR* genotyping for various reasons. TTG of other genes was not performed due to Korea’s health insurance coverage policy.

### cfDNA and tissue genomic DNA extraction

cfDNA was extracted from aliquots (500 µl) of serum using the QIAamp circulating nucleic acid kit (Qiagen, Hilden, Germany) with the QIAvac 24 Plus vacuum manifold, following the manufacturer’s instructions. cfDNA purity was checked using an Agilent High Sensitivity DNA Kit and the Bioanalyzer 2100 instrument (Agilent Technologies, Santa Clara, CA). When required, additional purification was performed using Agencourt AMPure XP (BeckMan Coulter, Brea, CA) to remove larger contaminating nucleic acid. cfDNA concentration was quantified with a Qubit 2.0 Fluorometer using the Agilent High Sensitivity DNA Kit (Agilent Technologies).

Tissue genomic DNA (gDNA) was extracted from formalin-fixed, paraffin-embedded (FFPE) tissues with the QIAamp DNA FFPE Tissue kit (Qiagen) according to the manufacturer’s instructions and eluted in a 50 μL volume. Purity of the extracted genomic DNA was assessed by electrophoresis of the DNA through a 1% agarose gel, and DNA concentration was quantified with a Qubit 2.0 Fluorometer using the Agilent High Sensitivity DNA Kit (Agilent Technologies).

### Genomic DNA extraction from buffy coat

gDNA was extracted from buffy coat using the MG blood genomic DNA extraction kit (MGmed, Seoul, Korea) following the manufacturer’s instructions. gDNA quantity and purity were measured using a Nanodrop 1000 (Thermo Fisher Scientific, Waltham, MA).

### Next-generation sequencing (NGS)

Overall, up to 10 ng of cfDNA and gDNA was extracted from serum, FFPE, and buffy coat and amplified using the Ion AmpliSeq™ Library Kit 2.0 (Life Technologies, Carlsbad, CA), with barcoding of each sample. Twenty cycles were performed. Library concentration was evaluated with QuantStudio™ Real-Time PCR Systems (Thermo Fisher Scientific). Each diluted library (100 pM) was amplified through emulsion PCR using the OneTouch™ Instrument (Life Technologies) and enriched by the OneTouch™ ES Instrument (Life Technologies) using the Ion PI Hi-Q OT2 200 kit following the manufacturer’s instructions. Finally, sequencing was performed on an Ion Proton instrument (Life Technologies) using an Ion PI Hi-Q Sequencing 200 Kit (Life Technologies). Barcoded samples were loaded onto an Ion PI Chip v3.

### Sequencing data analysis

Sequencing read mapping and variant calling were performed with Ion Torrent Suite v5.0.4.0. Because ultra-high depth sequencing is likely to produce many mismatched base-pairs due to the intrinsic chance of sequencing error, we controlled for this as follows: (1) we extracted RO (reference allele observation) and AO (alternate allele observation) values for each variant, (2) assuming that the sequencing error rate was 0.1% and following a *Poisson* distribution, we estimated the probability (*p*-value < 0.01) that the number of reads with the alternate allele was observed for each variant, (3) a variant frequency > 0.1% was selected for each sample. To determine the accuracy and minimum variant frequency threshold, we used the Multiplex I cfDNA Reference Standard Set (Horizon Discovery, Cambridge, MA). Because the AmpliSeq method is known to have some technical artifacts such as homopolymer indels [37], we sequenced available buffy coat samples (*n* = 4; #086, #092, #100, #109). Variants discovered from at least one of the four buffy coat samples were removed from the initial list of serum variants.

### Variant annotation and pathogenic variant definition

Variants were annotated with SnpEff (v4.1) [38] according to the genomic coordinates GRCh37.75. Then we evaluated if the variants were present in the dbSNP (v142) common database. Variants not found in the dbSNP database were further annotated with the ClinVar (20150804) database [39]. Pathogenic variants were annotated as “likely-pathogenic,” “pathogenic,” or “drug response” by the ClinVar database.

### Droplet digital PCR

Mutant allele frequency was assessed using the QX200 Droplet Digital PCR (ddPCR) System (BioRad, Milan, Italy) in accordance with the manufacturer’s instructions. The PrimePCR™ ddPCR™ Mutation Assay for humans was used. This kit evaluates *EGFR* p.E746_A750del and *EGFR* WT for p.E746_A750del, *EGFR* p.T790M and *EGFR* WT for p.T790M, *EGFR* p.L858R and *EGFR* WT for p.L858R, *KRAS* G12X and *KRAS* WT for G12X, *KRAS* G13X and *KRAS* WT for G13X, and *KRAS* Q61X and *KRAS* WT for Q61X. ddPCR reaction mixtures contained a final concentration of 250 nM of each of the probes, 450 nM of forward and reverse primers, 1x ddPCR Supermix for Probes (Bio-Rad), and 5∼50 ng DNA in a final volume of 20 µl. Each reaction included a blank sample corresponding to H_2_O, another corresponding to wild-type DNA, and a positive control. Fluorescence signals of blank and negative control samples were considered background and used to set up the cut-off. Entire ddPCR reaction volumes were loaded in the appropriate wells of a DG8 cartridge (Bio-Rad) with 70 µl of generator oil (Bio-Rad). Samples were then partitioned into approximately 20,000 water-oil emulsion droplets using the QX200 Droplet generator (Bio-Rad). Forty microliters of the water-oil emulsion were used for the ddPCR reaction that was performed with a C1000 Thermal cycler (Bio-Rad) under the following conditions: 1 cycle of 95°C for 10 min, 40 cycles of 94°C for 30 s and 55°C for 1 min, and 1 cycle of 98°C for 10 min. After thermal cycling, the plates were transferred to a QX200 Droplet reader. Digital PCR data were analyzed using QuantaSoft analytical software v1.7.4 (Bio-Rad).

## SUPPLEMENTARY MATERIALS FIGURE AND TABLES








