# Supplementary material for: Detection of somatic variants and EGFR mutations in cell-free DNA from non-small cell lung cancer patients by ultra-deep sequencing using the ion ampliseq cancer hotspot panel and droplet digital polymerase chain reaction
Source: Oncotarget. 2017 Nov 15;8(63):106901–12. doi: 10.18632/oncotarget.22456 (PMC5739783; doi:10.18632/oncotarget.22456)
Supplement: Supplementary file 1 [file oncotarget-08-106901-s001.pdf]

Detection of somatic variants and *EGFR* mutations in cell-free DNA from non-small cell lung cancer patients by ultra-deep sequencing using the ion ampliseq cancer hotspot panel and droplet digital polymerase chain reaction

SUPPLEMENTARY MATERIALS

Supplementary Table 1: Mutation detection accuracy of ICP and ddPCR using reference cfDNA

Reference cfDNA		ICP				ddPCR			
		0	5%	1%	0.1%	0	5%	1%	0.1%
EGFR	p.E746_A750del	0	7.82	1.54	0.21	0	7.51	1.23	0.14
	p.T790M	5/10043 (0.05)	5.52	0.71	0.25	0	4.40	0.96	0.17
	p.L858R	2/17844 (0.01)	4.10	0.28	0.08	0	5.26	1.02	0.10
KRAS	G12D	3/13805 (0.02)	5.99	0.88	0.07	0	7.84	4.07	0.33
NRAS	Q61K	6/33744 (0.02)	5.63	0.69	0.11				
PIK3CA	E545K	4/18620 (0.02)	6.81	1.46	0.19				

Supplementary Table 2A: Details of variants detected from 123 cfDNA using ultra-deep sequencing ICP. See Supplementary_Table_2A

Supplementary Table 2B: Number of variants according to the cell type

Variants (*n*)	Overall (123)	Adc (93)	Sqc (20)	Others (10)
0	12	7	4	1
1	15	8	6	1
2~10	81	66	9	6
11~20	10	9	0	1
21~40	3	2	1	0
100~	2	1	0	1
Average	6.93	6.83	3.85	14.1
Median	4	4	2	3

Supplementary Table 3: *EGFR* mutational screening using the Ion ampliSeq cancer hotspot panel (ICP) in cfDNA samples from 123 patients with lung cancer. See Supplementary_Table_3

Supplementary Table 4: *EGFR* mutations (including low-frequency mutations) in cfDNA based on ICP in 56 patients who were treated with an EGFR TKI. See Supplementary_Table_4

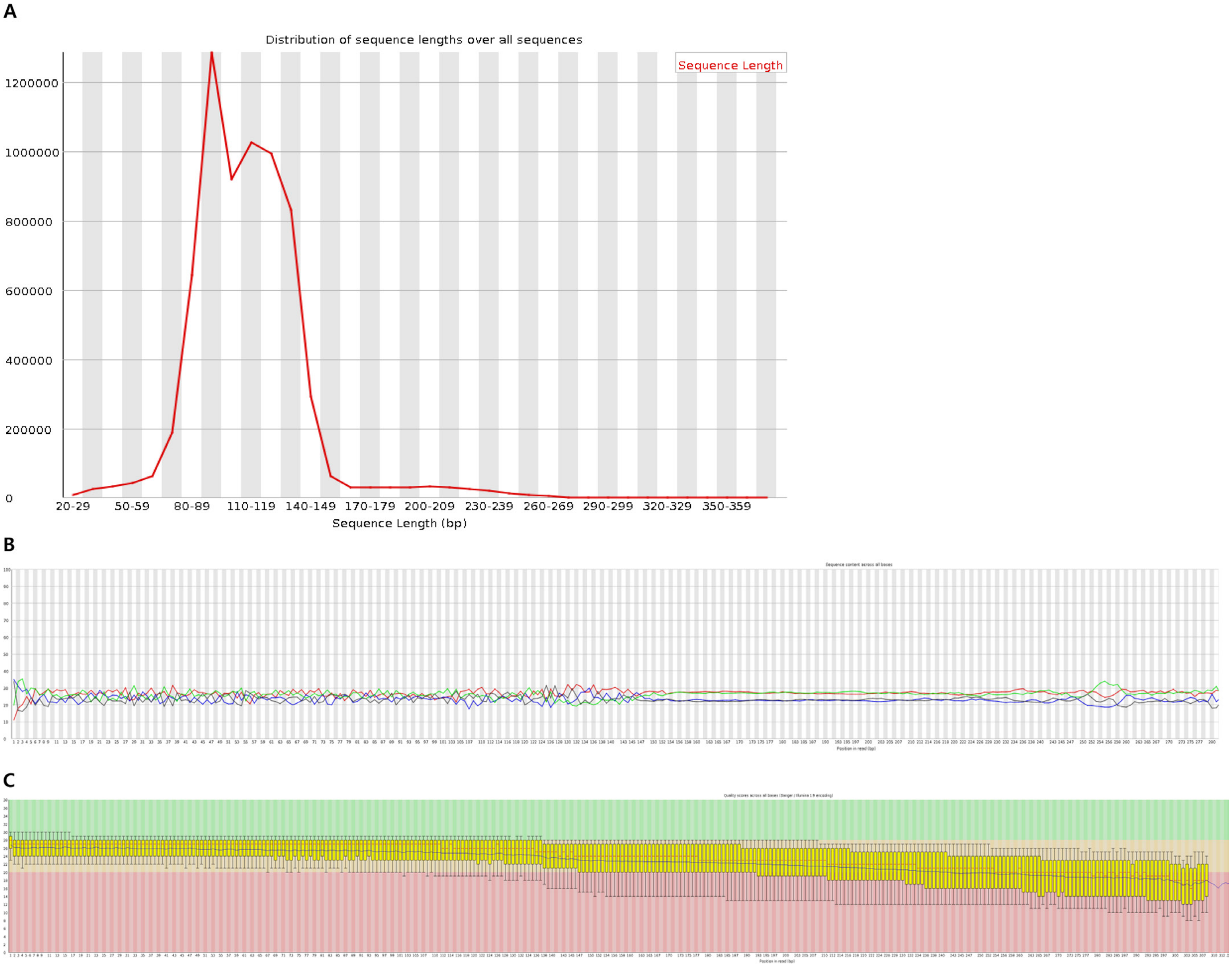

Supplementary Figure 1: Sequencing coverage. (**A**) Distribution of sequence lengths over all sequences of cfDNA. The most sequence lengths are between 60 to 170 bp. (**B**) GC content across all bases of cfDNA. (**C**) Quality scores across all bases of cfDNA.
