# Supplementary material for: Detection of somatic variants and EGFR mutations in cell-free DNA from non-small cell lung cancer patients by ultra-deep sequencing using the ion ampliseq cancer hotspot panel and droplet digital polymerase chain reaction
Source: Oncotarget. 2017 Nov 15;8(63):106901–12. doi: 10.18632/oncotarget.22456 (PMC5739783; doi:10.18632/oncotarget.22456)
Supplement: Supplementary file 2 [file oncotarget-08-106901-s002.docx]

**Supplementary Table 2A: Details of variants detected from 123 cfDNA using ultra-deep sequencing ICP**

| Gene | HGVS.protein | Overall (123) | Adc (93) | Sqc (20) | Others (10) |
| --- | --- | --- | --- | --- | --- |
| TP53 | p.Arg273His | 41 | 34 | 4 | 3 |
| TP53 | p.Met237Val | 37 | 29 | 6 | 2 |
| TP53 | p.Tyr234Cys | 35 | 29 | 5 | 1 |
| IDH2 | p.Arg140Gln | 33 | 23 | 5 | 5 |
| EGFR | p.Leu858Arg | 30 | 26 | 1 | 3 |
| EGFR | p.Thr790Met | 30 | 24 | 3 | 3 |
| PIK3CA | p.His1047Arg | 24 | 18 | 4 | 2 |
| PTEN | p.His61Arg | 21 | 17 | 2 | 2 |
| PIK3CA | p.Cys420Arg | 14 | 14 | 0 | 0 |
| BRAF | p.Phe595Leu | 14 | 11 | 3 | 0 |
| TP53 | p.Arg282Trp | 14 | 10 | 1 | 3 |
| HRAS | p.Gly12Ser | 13 | 9 | 2 | 2 |
| EGFR | p.Leu747_Glu749del | 12 | 10 | 1 | 1 |
| TP53 | p.Ala189Val | 12 | 9 | 1 | 2 |
| TP53 | p.Arg175His | 12 | 8 | 2 | 2 |
| MET | p.Tyr1248Cys | 11 | 10 | 1 | 0 |
| TP53 | p.Tyr163Cys | 10 | 10 | 0 | 0 |
| NRAS | p.Gly12Asp | 10 | 9 | 0 | 1 |
| TP53 | p.Ala161Thr | 10 | 9 | 0 | 1 |
| RET | p.Cys634Tyr | 10 | 8 | 1 | 1 |
| TP53 | p.Arg273Cys | 9 | 7 | 1 | 1 |
| VHL | p.Arg161* | 9 | 7 | 1 | 1 |
| TP53 | p.Arg283His | 9 | 7 | 0 | 2 |
| TP53 | p.Arg181His | 9 | 6 | 2 | 1 |
| TP53 | p.Arg158His | 9 | 6 | 1 | 2 |
| TP53 | p.Arg248Gln | 8 | 6 | 1 | 1 |
| TP53 | p.Cys176Tyr | 8 | 6 | 1 | 1 |
| TP53 | p.Arg267Trp | 8 | 5 | 1 | 2 |
| TP53 | p.Arg248Trp | 8 | 5 | 1 | 2 |
| VHL | p.Arg167Gln | 8 | 5 | 1 | 2 |
| KRAS | p.Gly13Gly | 7 | 6 | 0 | 1 |
| RB1 | p.Arg455* | 7 | 6 | 0 | 1 |
| EGFR | p.Glu746_Ala750del | 7 | 6 | 0 | 1 |
| TP53 | p.Cys242Tyr | 7 | 5 | 1 | 1 |
| TP53 | p.Gln165* | 7 | 5 | 1 | 1 |
| IDH1 | p.Arg132His | 7 | 5 | 1 | 1 |
| TP53 | p.Arg181Cys | 7 | 5 | 0 | 2 |
| SMAD4 | p.Arg361His | 7 | 4 | 1 | 2 |
| GNAS | p.Arg844His | 7 | 4 | 1 | 2 |
| EGFR | p.Glu746_Ala750del | 6 | 6 | 0 | 0 |
| KRAS | p.Gly12Ser | 6 | 4 | 1 | 1 |
| SRC | p.Gln537* | 6 | 4 | 1 | 1 |
| TP53 | p.Pro152Leu | 6 | 3 | 1 | 2 |
| APC | p.Gln1096* | 6 | 3 | 1 | 2 |
| NRAS | p.Gly13Asp | 5 | 4 | 1 | 0 |
| TP53 | p.Arg342* | 5 | 4 | 0 | 1 |
| PTEN | p.Gln245* | 5 | 3 | 1 | 1 |
| TP53 | p.Cys277Tyr | 5 | 3 | 1 | 1 |
| KIT | p.Glu839Lys | 5 | 3 | 1 | 1 |
| PTEN | p.Tyr68His | 5 | 3 | 0 | 2 |
| TP53 | p.Arg213* | 5 | 3 | 0 | 2 |
| TP53 | p.Ser127Phe | 5 | 3 | 0 | 2 |
| KRAS | p.Gly12Cys | 4 | 4 | 0 | 0 |
| KIT | p.Val559Ala | 4 | 3 | 1 | 0 |
| PTEN | p.Arg173Cys | 4 | 3 | 0 | 1 |
| TP53 | p.Arg283Cys | 4 | 3 | 0 | 1 |
| TP53 | p.Gly245Asp | 4 | 3 | 0 | 1 |
| SMAD4 | p.Arg445* | 4 | 3 | 0 | 1 |
| PTEN | p.Pro246Leu | 4 | 2 | 1 | 1 |
| PTEN | p.Gln261* | 4 | 2 | 1 | 1 |
| HRAS | p.Gly12Asp | 4 | 2 | 1 | 1 |
| TP53 | p.Arg337Cys | 4 | 2 | 1 | 1 |
| ERBB2 | p.Gly776Ser | 4 | 2 | 1 | 1 |
| VHL | p.Arg167Trp | 4 | 2 | 1 | 1 |
| TP53 | p.Gly245Ser | 4 | 2 | 0 | 2 |
| PTEN | p.Leu112Pro | 3 | 3 | 0 | 0 |
| KIT | p.Asp816Val | 3 | 3 | 0 | 0 |
| EGFR | p.Leu747_Thr751del | 3 | 3 | 0 | 0 |
| BRAF | p.Val600Glu | 3 | 3 | 0 | 0 |
| RET | p.Cys618Arg | 3 | 2 | 1 | 0 |
| PTEN | p.Gln214* | 3 | 2 | 0 | 1 |
| HRAS | p.Gly13Asp | 3 | 2 | 0 | 1 |
| ATM | p.Arg3047* | 3 | 2 | 0 | 1 |
| KRAS | p.Gln61Lys | 3 | 2 | 0 | 1 |
| KRAS | p.Thr58Ile | 3 | 2 | 0 | 1 |
| KRAS | p.Gly13Asp | 3 | 2 | 0 | 1 |
| PTPN11 | p.Glu76Lys | 3 | 2 | 0 | 1 |
| AKT1 | p.Glu17Lys | 3 | 2 | 0 | 1 |
| TP53 | p.Arg337His | 3 | 2 | 0 | 1 |
| TP53 | p.Arg306* | 3 | 2 | 0 | 1 |
| TP53 | p.Val216Met | 3 | 2 | 0 | 1 |
| STK11 | p.Gln170* | 3 | 2 | 0 | 1 |
| VHL | p.Pro86Ser | 3 | 2 | 0 | 1 |
| FGFR3 | p.Arg248Cys | 3 | 2 | 0 | 1 |
| FIP1L1 | p.Thr434Ile | 3 | 2 | 0 | 1 |
| EGFR | p.Thr751Ile | 3 | 2 | 0 | 1 |
| SMO | p.Trp535Leu | 3 | 2 | 0 | 1 |
| BRAF | p.Asp594Asn | 3 | 2 | 0 | 1 |
| JAK2 | p.Val617Phe | 3 | 2 | 0 | 1 |
| PIK3CA | p.Glu542Lys | 3 | 1 | 1 | 1 |
| APC | p.Arg1450* | 3 | 1 | 1 | 1 |
| TP53 | p.Tyr220Cys | 3 | 1 | 0 | 2 |
| PTEN | p.Gln17* | 2 | 2 | 0 | 0 |
| PTEN | p.His123Tyr | 2 | 2 | 0 | 0 |
| KRAS | p.Gly12Asp | 2 | 2 | 0 | 0 |
| PTPN11 | p.Pro491Leu | 2 | 2 | 0 | 0 |
| TP53 | p.Met237Ile | 2 | 2 | 0 | 0 |
| TP53 | p.His179Tyr | 2 | 2 | 0 | 0 |
| MPL | p.Ser505Asn | 2 | 1 | 1 | 0 |
| PTEN | p.Arg130Gln | 2 | 1 | 0 | 1 |
| PTEN | p.Gln171* | 2 | 1 | 0 | 1 |
| PTEN | p.Arg335* | 2 | 1 | 0 | 1 |
| HRAS | p.Gly13Cys | 2 | 1 | 0 | 1 |
| ATM | p.Arg3008His | 2 | 1 | 0 | 1 |
| KRAS | p.Val14Ile | 2 | 1 | 0 | 1 |
| PTPN11 | p.Asp61Asn | 2 | 1 | 0 | 1 |
| PTPN11 | p.Ser502Leu | 2 | 1 | 0 | 1 |
| RB1 | p.Arg358* | 2 | 1 | 0 | 1 |
| RB1 | p.Arg552* | 2 | 1 | 0 | 1 |
| RB1 | p.Arg556* | 2 | 1 | 0 | 1 |
| RB1 | p.Arg579* | 2 | 1 | 0 | 1 |
| RB1 | p.Glu748* | 2 | 1 | 0 | 1 |
| TP53 | p.Glu286Lys | 2 | 1 | 0 | 1 |
| TP53 | p.Ser241Phe | 2 | 1 | 0 | 1 |
| TP53 | p.Cys238Tyr | 2 | 1 | 0 | 1 |
| TP53 | p.Arg196* | 2 | 1 | 0 | 1 |
| TP53 | p.His193Arg | 2 | 1 | 0 | 1 |
| STK11 | p.Asp194Asn | 2 | 1 | 0 | 1 |
| STK11 | p.Asp194Tyr | 2 | 1 | 0 | 1 |
| SMARCB1 | p.Arg386His | 2 | 1 | 0 | 1 |
| VHL | p.Pro86Leu | 2 | 1 | 0 | 1 |
| CTNNB1 | p.Asp32Tyr | 2 | 1 | 0 | 1 |
| PIK3CA | p.Glu545Lys | 2 | 1 | 0 | 1 |
| PIK3CA | p.His1047Tyr | 2 | 1 | 0 | 1 |
| FIP1L1 | p.Asp606Tyr | 2 | 1 | 0 | 1 |
| EGFR | p.Glu709Lys | 2 | 1 | 0 | 1 |
| BRAF | p.Thr599Ile | 2 | 1 | 0 | 1 |
| NRAS | p.Gly60Glu | 1 | 1 | 0 | 0 |
| PTEN | p.Tyr16fs | 1 | 1 | 0 | 0 |
| PTEN | p.Gly129Arg | 1 | 1 | 0 | 0 |
| PTEN | p.Gln298* | 1 | 1 | 0 | 0 |
| ATM | p.Arg3008Cys | 1 | 1 | 0 | 0 |
| KRAS | p.Gln61His | 1 | 1 | 0 | 0 |
| KRAS | p.Ala59Thr | 1 | 1 | 0 | 0 |
| KRAS | p.Gly12Val | 1 | 1 | 0 | 0 |
| PTPN11 | p.Asp61Val | 1 | 1 | 0 | 0 |
| PTPN11 | p.Thr73Ile | 1 | 1 | 0 | 0 |
| PTPN11 | p.Gly503Glu | 1 | 1 | 0 | 0 |
| FLT3 | p.Asp835Tyr | 1 | 1 | 0 | 0 |
| FLT3 | p.Asp835Asn | 1 | 1 | 0 | 0 |
| RB1 | p.Glu137* | 1 | 1 | 0 | 0 |
| RB1 | p.Arg320* | 1 | 1 | 0 | 0 |
| CDH1 | p.Thr340Ala | 1 | 1 | 0 | 0 |
| TP53 | p.Arg337Leu | 1 | 1 | 0 | 0 |
| TP53 | p.Glu298* | 1 | 1 | 0 | 0 |
| TP53 | p.Arg282Gly | 1 | 1 | 0 | 0 |
| TP53 | p.Gly245Cys | 1 | 1 | 0 | 0 |
| TP53 | p.Arg213Gln | 1 | 1 | 0 | 0 |
| GNAS | p.Arg844Ser | 1 | 1 | 0 | 0 |
| VHL | p.Trp88* | 1 | 1 | 0 | 0 |
| CTNNB1 | p.Ser33Tyr | 1 | 1 | 0 | 0 |
| CTNNB1 | p.Gly34Glu | 1 | 1 | 0 | 0 |
| CTNNB1 | p.Thr41Ile | 1 | 1 | 0 | 0 |
| PIK3CA | p.Met1043Ile | 1 | 1 | 0 | 0 |
| FGFR3 | p.Gly382Arg | 1 | 1 | 0 | 0 |
| FGFR3 | p.Ala393Glu | 1 | 1 | 0 | 0 |
| KIT | p.Asp816Tyr | 1 | 1 | 0 | 0 |
| EGFR | p.Glu746_Glu749del | 1 | 1 | 0 | 0 |
| EGFR | p.Glu746_Thr751delinsAla | 1 | 1 | 0 | 0 |
| BRAF | p.Val600Gly | 1 | 1 | 0 | 0 |
| BRAF | p.Phe595Leu | 1 | 1 | 0 | 0 |
| BRAF | p.Gly466Val | 1 | 1 | 0 | 0 |
| BRAF | p.Gly464Val | 1 | 1 | 0 | 0 |
| BRAF | p.Arg462Ile | 1 | 1 | 0 | 0 |
| TP53 | p.Arg249Ser | 1 | 0 | 1 | 0 |
| ABL1 | p.Tyr272His | 1 | 0 | 0 | 1 |
