# Supplementary material for: Detection of somatic variants and EGFR mutations in cell-free DNA from non-small cell lung cancer patients by ultra-deep sequencing using the ion ampliseq cancer hotspot panel and droplet digital polymerase chain reaction
Source: Oncotarget. 2017 Nov 15;8(63):106901–12. doi: 10.18632/oncotarget.22456 (PMC5739783; doi:10.18632/oncotarget.22456)
Supplement: Supplementary file 3 [file oncotarget-08-106901-s003.docx]

**Supplementary Table 3: *EGFR* mutational screening using the Ion AmpliSeq Cancer Hotspot Panel (ICP) in cfDNA samples from 123 patients with lung cancer**

|  |  |  |  | ***EGFR* activating mutations status** | | | | |  |  |  |
| --- | --- | --- | --- | --- | --- | --- | --- | --- | --- | --- | --- |
| **Patient No.** | **Cell type** | **Tissue-blood duration (days)** | **Variant No.** | **Exon 19 deletion** | |  | **L858R** | |  | **T790M** | |
|  |  |  |  | **TTG** | **ICP (%)** |  | **TTG** | **ICP (%)** |  | **TTG** | **ICP (%)** |
| #001 | Adc | 3 | 3 | W | W |  | W | W |  | W | W |
| #002 | Adc | 6 | 13 | W | Mut (0.318) |  | W | W |  | W | W |
| #003 | Adc | 10 | 6 | W | Mut (0.691) |  | W | W |  | W | W |
| #004 | Adc | 4 | 1 | Mut | W |  | W | W |  | W | W |
| #005 | Sqcc | 37 | 1 | W | W |  | W | W |  | W | W |
| #006 | Adc | 6 | 7 | W | Mut (0.247) |  | W | Mut (0.226) |  | W | W |
| #007 | Adc | 5 | 3 | W | W |  | W | W |  | W | W |
| #008 | Adc | 0 | 1 | W | W |  | W | W |  | W | W |
| #009 | Others | 1 | 3 | Mut | W |  | W | W |  | W | W |
| #010 | Adc | 7 | 6 | W | W |  | W | Mut (0.322) |  | W | W |
| #011 | Sqcc | 16 | 5 | W | W |  | W | W |  | W | W |
| #012 | Sqcc | 3 | 4 | W | W |  | W | W |  | W | W |
| #013 | Sqcc | 0 | 2 | W | W |  | W | W |  | W | W |
| #014 | Adc | 1 | 2 | W | W |  | W | W |  | W | W |
| #015 | Others | 4 | 3 | W | W |  | W | W |  | W | W |
| #016 | Sqcc | 0 | 6 | W | W |  | W | W |  | W | W |
| #017 | Adc | 4 | 9 | W | W |  | W | Mut (0.189) |  | W | W |
| #018 | Adc | 0 | 0 | W | W |  | W | W |  | W | W |
| #019 | Sqcc | 3 | 0 | W | W |  | W | W |  | W | W |
| #020 | Others | 3 | 0 | W | W |  | W | W |  | W | W |
| #021 | Adc | 2 | 1 | W | W |  | W | W |  | W | W |
| #022 | Adc | 0 | 3 | W | W |  | W | W |  | W | W |
| #023 | Sqcc | 4 | 1 | W | W |  | W | W |  | W | W |
| #024 | Adc | 17 | 5 | W | W |  | W | W |  | W | W |
| #025 | Adc | 0 | 2 | W | W |  | W | W |  | W | W |
| #026 | Others | 0 | 3 | W | Mut (7.413) |  | W | W |  | W | W |
| #027 | Adc | 7 | 0 | W | W |  | W | W |  | W | W |
| #028 | Adc | 0 | 10 | W | W |  | W | W |  | W | W |
| #029 | Adc | 1 | 1 | W | W |  | W | W |  | W | W |
| #030 | Others | 9 | 2 | W | W |  | W | W |  | W | W |
| #031 | Adc | 1 | 0 | W | W |  | W | W |  | W | W |
| #032 | Sqcc | 0 | 1 | W | W |  | Mut | W |  | W | W |
| #033 | Adc | 6 | 2 | W | W |  | W | W |  | W | W |
| #034 | Adc | 6 | 0 | W | W |  | W | W |  | W | W |
| #035 | Adc | 365 | 2 | W | W |  | W | W |  | W | W |
| #036 | Sqcc | 0 | 0 | W | W |  | W | W |  | W | W |
| #037 | Sqcc | 0 | 1 | W | W |  | W | W |  | W | W |
| #038 | Adc | 0 | 3 | W | W |  | Mut | Mut (28.489) |  | W | W |
| #039 | Sqcc | 1 | 1 | W | W |  | W | W |  | W | W |
| #040 | Adc | 1 | 1 | W | W |  | Mut | W |  | W | W |
| #041 | Adc | 22 | 2 | Mut | Mut (0.200) |  | W | W |  | W | W |
| #042 | Adc | no tissue | 1 | ND | W |  | ND | W |  | ND | W |
| #043 | Others | no tissue | 1 | ND | W |  | ND | W |  | ND | W |
| #044 | Adc | 2 | 9 | W | W |  | W | W |  | W | W |
| #045 | Sqcc | no tissue | 33 | ND | W |  | ND | W |  | ND | W |
| #046 | Adc | 16 | 2 | Mut | Mut (1.325) |  | W | W |  | W | W |
| #047 | Adc | 3 | 2 | W | W |  | W | Mut (2.103) |  | W | W |
| #048 | Adc | 7 | 4 | Mut | W |  | W | W |  | W | W |
| #049 | Adc | no tissue | 0 | ND | W |  | ND | W |  | ND | W |
| #050 | Adc | no tissue | 10 | ND | W |  | ND | Mut (2.647) |  | ND | Mut (2.416) |
| #051 | Adc | 33 | 8 | Mut | W |  | W | Mut (2.099) |  | W | Mut (0.443) |
| #052 | Sqcc | no tissue | 7 | ND | W |  | ND | Mut (0.360) |  | ND | Mut (0.617) |
| #053 | Adc | no tissue | 4 | ND | W |  | ND | W |  | ND | W |
| #054 | Adc | no tissue | 2 | ND | W |  | ND | W |  | ND | W |
| #055 | Adc | no tissue | 32 | ND | W |  | ND | W |  | ND | W |
| #056 | Adc | no tissue | 2 | ND | W |  | ND | W |  | ND | W |
| #057 | Adc | 1144 | 1 | Mut | W |  | W | W |  | W | W |
| #058 | Adc | no tissue | 7 | ND | W |  | ND | W |  | ND | W |
| #059 | Adc | 14 | 4 | W | Mut (0.684) |  | W | W |  | W | W |
| #060 | Sqcc | no tissue | 4 | ND | W |  | ND | W |  | ND | Mut (2.895) |
| #061 | Adc | 0 | 2 | Mut | W |  | W | W |  | W | W |
| #062 | Adc | 3 | 2 | W | W |  | Mut | Mut (0.304) |  | W | W |
| #063 | Adc | 4 | 5 | W | W |  | W | Mut (0.680) |  | W | Mut (0.874) |
| #064 | Adc | 2 | 6 | W | W |  | W | W |  | W | W |
| #065 | Adc | 5 | 3 | W | W |  | W | W |  | W | W |
| #066 | Adc | 6 | 4 | W | W |  | W | W |  | W | Mut (0.298) |
| #067 | Sqcc | 16 | 4 | W | W |  | W | W |  | W | Mut (0.369) |
| #068 | Adc | 3 | 2 | W | W |  | W | W |  | W | W |
| #069 | Adc | no tissue | 5 | ND | W |  | ND | Mut (0.313) |  | ND | Mut (0.569) |
| #070 | Sqcc | no tissue | 2 | ND | W |  | ND | W |  | ND | W |
| #071 | Adc | 5 | 125 | W | W |  | W | Mut (0.451) |  | W | Mut (0.684) |
| #072 | Adc | 1239 | 18 | W | W |  | Mut | Mut (0.520) |  | W | Mut (3.191) |
| #073 | Adc | 7 | 5 | W | W |  | W | Mut (0.653) |  | W | Mut (2.063) |
| #074 | Adc | no tissue | 5 | ND | W |  | ND | Mut (1.357) |  | ND | Mut (1.283) |
| #075 | Adc | 11 | 3 | W | W |  | W | W |  | W | W |
| #076 | Adc | 13 | 39 | Mut | Mut (0.180) |  | W | Mut (0.162) |  | W | Mut (0.417) |
| #077 | Adc | 5 | 13 | W | W |  | W | Mut (1.083) |  | W | Mut (1.240) |
| #078 | Others | no tissue | 13 | ND | W |  | ND | Mut (0.247) |  | ND | Mut (0.226) |
| #079 | Adc | no tissue | 2 | ND | W |  | ND | W |  | ND | W |
| #080 | Sqcc | no tissue | 0 | ND | W |  | ND | W |  | ND | W |
| #081 | Others | 24 | 9 | W | W |  | W | Mut (0.585) |  | W | Mut (0.606) |
| #082 | Adc | no tissue | 12 | ND | W |  | ND | W |  | ND | Mut (0.220) |
| #083 | Others | no tissue | 101 | ND | W |  | ND | Mut (0.163) |  | ND | Mut (10.308) |
| #084 | Adc | 327 | 8 | W | W |  | Mut | Mut (0.273) |  | W | Mut (1.105) |
| #085 | Adc | 5 | 5 | W | W |  | W | W |  | W | Mut (0.502) |
| #086 | Adc | 1 | 11 | W | W |  | Mut | Mut (0.211) |  | W | Mut (9.559) |
| #087 | Adc | 10 | 14 | Mut | Mut (44.819) |  | W | Mut (3.055) |  | W | Mut (1.480) |
| #088 | Adc | 5 | 9 | W | W |  | W | W |  | W | Mut (0.238) |
| #089 | Adc | 40 | 9 | W | W |  | W | W |  | W | Mut (0.300) |
| #090 | Adc | 6 | 7 | W | W |  | W | Mut (0.245) |  | W | Mut (0.407) |
| #091 | Adc | 7 | 7 | W | W |  | W | W |  | W | W |
| #092 | Adc | 6 | 3 | W | W |  | W | W |  | W | W |
| #093 | Adc | 14 | 3 | Mut | W |  | W | W |  | W | W |
| #094 | Adc | 2 | 0 | W | W |  | W | W |  | W | W |
| #095 | Sqcc | 1 | 0 | W | W |  | W | W |  | W | W |
| #096 | Adc | no tissue | 7 | ND | Mut (3.110) |  | ND | W |  | ND | W |
| #097 | Adc | 6 | 1 | W | W |  | W | W |  | W | W |
| #098 | Adc | 11 | 0 | Mut | W |  | W | W |  | W | W |
| #099 | Adc | 1 | 9 | W | W |  | W | Mut (0.627) |  | W | Mut (0.226) |
| #100 | Adc | 5 | 6 | W | W |  | W | Mut (0.211) |  | W | W |
| #101 | Adc | 0 | 3 | W | Mut (2.068) |  | Mut | Mut (2.339) |  | W | W |
| #102 | Sqcc | no tissue | 1 | ND | Mut (1.642) |  | ND | W |  | ND | W |
| #103 | Adc | 1 | 2 | W | Mut (0.672) |  | W | W |  | W | W |
| #104 | Others | 0 | 6 | W | Mut (0.727) |  | W | W |  | W | W |
| #105 | Adc | 2 | 3 | W | W |  | W | W |  | W | W |
| #106 | Sqcc | 0 | 4 | W | W |  | W | W |  | W | W |
| #107 | Adc | 1 | 7 | Mut | W |  | W | W |  | W | W |
| #108 | Adc | 6 | 5 | Mut | Mut (1.612) |  | W | W |  | W | W |
| #109 | Adc | no tissue | 11 | ND | W |  | ND | W |  | ND | W |
| #110 | Adc | 669 | 3 | Mut | Mut (2.720) |  | W | W |  | W | Mut (0.973) |
| #111 | Adc | 11 | 4 | W | W |  | Mut | W |  | W | W |
| #112 | Adc | 26 | 4 | Mut | W |  | W | W |  | W | W |
| #113 | Adc | 2 | 2 | Mut | W |  | W | W |  | W | W |
| #114 | Adc | 63 | 16 | W | Mut (1.037) |  | W | W |  | W | W |
| #115 | Adc | 10 | 13 | Mut | Mut (1.416) |  | W | W |  | W | W |
| #116 | Adc | 14 | 9 | W | Mut (3.172) |  | Mut | Mut (2.315) |  | W | W |
| #117 | Adc | 14 | 6 | W | Mut (1.077) |  | Mut | W |  | W | Mut (1.557) |
| #118 | Adc | 26 | 6 | Mut | Mut (7.362) |  | W | W |  | W | Mut (0.239) |
| #119 | Adc | 9 | 8 | Mut | Mut (3.341) |  | W | Mut (0.196) |  | W | W |
| #120 | Adc | 20 | 4 | W | Mut (0.393) |  | Mut | W |  | W | Mut (0.190) |
| #121 | Adc | 9 | 2 | Mut | W |  | W | W |  | W | 0 |
| #122 | Adc | 5 | 2 | W | W |  | W | W |  | W | W |
| #123 | Adc | 9 | 4 | W | Mut (0.539) |  | Mut | Mut (0.206) |  | W | W |

W: Wild genotype; Mut: Mutant genotype; ND: Not done.
