# Supplementary material for: Detection of somatic variants and EGFR mutations in cell-free DNA from non-small cell lung cancer patients by ultra-deep sequencing using the ion ampliseq cancer hotspot panel and droplet digital polymerase chain reaction
Source: Oncotarget. 2017 Nov 15;8(63):106901–12. doi: 10.18632/oncotarget.22456 (PMC5739783; doi:10.18632/oncotarget.22456)
Supplement: Supplementary file 4 [file oncotarget-08-106901-s004.docx]

**Supplementary Table 4: *EGFR* mutations (including low-frequency mutations) in cfDNA based on ICP in 56 patients who were treated with an EGFR TKI**

|  | ***EGFR* activating mutations status** | | | | | | |  |  |  |  |  |  |  |
| --- | --- | --- | --- | --- | --- | --- | --- | --- | --- | --- | --- | --- | --- | --- |
| **Patient No.** | **Exon 19 deletion** | | |  | **L858R** | | |  | **T790M** | | **TKI Tx** | **Response** | **TTP (months)** | **Comprehensive *EGFR* status** |
|  | **TTG** | **ICP (%)** | **ddPCR (%)** |  | **TTG** | **ICP (%)** | **ddPCR (%)** |  | **ICP (%)** | **ddPCR (%)** |  |  |  |  |
| #108 | Mut | Mut (1.612) | 3.700 | TP | W | W (0.030) | 0 | TN | W (0.068) | 0 | gefitinib | NE |  | Mut |
| #057 | Mut | W (0) | 0 | TN | W | W (0.023) | 0.005 | TP | W (0.049) | 0.039 | gefitinib | NE |  | Mut |
| #121 | Mut | W (0.120) | 39.000 | FN | W | W (0.004) | 0 | TN | W (0.060) | 0 | afatinib | NE |  | Mut |
| #071 | W | W (0) | ND | TN | W | Mut (0.451) | 0.045 | TP | Mut (0.684) | 0.060 | gefitinib | NE |  | Mut |
| #018 | W | W (0) | ND | TN | W | W (0.014) | 0 | TN | W (0.051) | 0 | gefitinib | NE |  | W |
| #024 | W | W (0) | ND | TN | W | W (0.013) | Failed | TN | W (0.106) | 0 | gefitinib | NE |  | W |
| #052 | ND | W (0) | ND | TN | ND | Mut (0.360) | 0 | FP | Mut (0.617) | 0.032 | erlotinib | NE |  | W |
| #082 | ND | W (0) | ND | TN | ND | W (0.066) | 0.032 | TP | Mut (0.220) | 0.090 | erlotinib | NE |  | Mut |
| #062 | W | W (0.006) | 0.016 | TP | Mut | Mut (0.304) | 0.710 | TP | W (0.143) | 0.035 | gefitinib | PD |  | Mut |
| #103 | W | Mut (0.672) | 0 | FP | W | W (0.010) | 0 | TN | W (0.061) | 0.016 | erlotinib | PD |  | W |
| #066 | W | W (0) | ND | TN | W | W (0.037) | 0 | TN | Mut (0.298) | 0 | erlotinib | PD |  | W |
| #023 | W | W (0) | ND | TN | W | W (0.014) | 0 | TN | W (0.069) | 0.018 | erlotinib | PD |  | W |
| #025 | W | W (0.094) | 0 | TN | W | W (0) | ND | TN | W (0.034) | 0 | erlotinib | PD |  | W |
| #029 | W | W (0) | ND | TN | W | W (0.014) | 0.280 | TP | W (0.061) | 0.015 | erlotinib | PD |  | Mut |
| #037 | W | W (0) | ND | TN | W | W (0) | ND | TN | W (0.021) | 0.070 | erlotinib | PD |  | W |
| #043 | ND | W (0) | ND | TN | ND | W (0.012) | 0 | TN | W (0.048) | 0.040 | erlotinib | PD |  | W |
| #045 | ND | W (0) | ND | TN | ND | W (0.020) | 0 | TN | W (0.139) | 0 | erlotinib | PD |  | W |
| #055 | ND | W (0) | ND | TN | ND | W (0.016) | 0 | TN | W (0.110) | 0.050 | erlotinib | PD |  | W |
| #056 | ND | W (0) | ND | TN | ND | W (0.066) | Failed | TN | W (0.064) | 0.060 | erlotinib | PD |  | W |
| #058 | ND | W (0.068) | ND | TN | ND | W (0.018) | 0 | TN | W (0) | ND | gefitinib | PD |  | W |
| #041 | Mut | Mut (0.200) | 0.160 | TP | W | W (0.022) | 0.017 | TN | W (0.038) | 0.024 | gefitinib | PR | 10 | Mut |
| #046 | Mut | Mut (1.325) | 2.410 | TP | W | W (0.058) | 0.060 | TP | W (0.060) | 0.060 | gefitinib | PR | 11 | Mut |
| #115 | Mut | Mut (1.416) | NA | TP | W | W (0) | NA | TN | W (0.078) | NA | afatinib | PR | 10+ | Mut |
| #117 | W | Mut (1.077) | Failed | NA | Mut | W (0.004) | Failed | NA | Mut (1.557) | 0 | erlotinib | PR | 8+ | Mut |
| #110 | Mut | Mut (2.720) | 4.480 | TP | W | W (0.024) | 0 | TN | Mut (0.973) | 1.270 | gefitinib | PR | 14 | Mut |
| #118 | Mut | Mut (7.362) | 17.200 | TP | W | W (0.009) | NA | NA | Mut (0.239) | Failed | afatinib | PR | 6 | Mut |
| #120 | W | Mut (0.393) | 0 | FP | Mut | W (0.008) | 0 | TP | Mut (0.190) | 0 | afatinib | PR | 19+ | Mut |
| #119 | Mut | Mut (3.341) | 0.800 | TP | W | Mut (0.196) | 0.120 | TP | W (0.044) | NA | afatinib | PR | 10+ | Mut |
| #101 | W | Mut (2.068) | 0 | FP | Mut | Mut (2.339) | 1.690 | TP | W (0) | ND | gefitinib | PR | 10+ | Mut |
| #123 | W | Mut (0.539) | 0 | FP | Mut | Mut (0.206) | Failed | TP | W (0.023) | NA | afatinib | PR | 8+ | Mut |
| #087 | Mut | Mut (44.819) | 58.000 | TP | W | Mut (3.055) | 0.024 | TP | Mut (1.480) | 0 | gefitinib | PR | 5 | Mut |
| #038 | W | W (0) | ND | TN | Mut | Mut (28.489) | 47.700 | TP | W (0.020) | 0.025 | gefitinib | PR | 4 | Mut |
| #051 | Mut | W (0) | 0 | TN | W | Mut (2.099) | 0 | FP | Mut (0.443) | 0.050 | gefitinib | PR | 76+ | Mut |
| #072 | W | W (0) | ND | TN | Mut | Mut (0.520) | 0 | TP | Mut (3.191) | 0.035 | gefitinib | PR | 9 | Mut |
| #084 | W | W (0) | ND | TN | Mut | Mut (0.273) | 0.070 | TP | Mut (1.105) | 0 | erlotinib | PR | 10 | Mut |
| #086 | W | W (0) | ND | TN | Mut | Mut (0.211) | 0.060 | TP | Mut (9.559) | 0.060 | gefitinib | PR | 16 | Mut |
| #048 | Mut | W (0.050) | 0.020 | FN | W | W (0.013) | 0.020 | TP | W (0.039) | 0.040 | gefitinib | PR | 66+ | Mut |
| #098 | Mut | W (0) | 0 | TN | W | W (0.007) | 0.008 | TP | W (0.066) | 0.021 | gefitinib | PR | 14+ | Mut |
| #107 | Mut | W (0) | 0 | TN | W | W (0.028) | 0.032 | TP | W (0.048) | 0.100 | erlotinib | PR | 8 | Mut |
| #112 | Mut | W (0) | 0.080 | FN | W | W (0.005) | 0 | TN | W (0.053) | NA | gefitinib | PR | 13 | Mut |
| #113 | Mut | W (0.048) | 0 | TN | W | W (0.024) | 0 | TN | W (0.037) | 0.030 | gefitinib | PR | 13+ | Mut |
| #111 | W | W (0) | ND | TN | Mut | W (0.005) | 0 | NA | W (0.053) | 0.060 | gefitinib | PR | 14+ | Mut |
| #026 | W | Mut (7.413) | 8.000 | TP | W | W (0.029) | 0 | TN | W (0) | ND | gefitinib | PR | 8 | Mut |
| #059 | W | Mut (0.684) | 5.600 | TP | W | W (0.047) | NA | NA | W (0.090) | NA | erlotinib | PR | 9 | Mut |
| #020 | W | W (0) | ND | TN | W | W (0.019) | 0 | TN | W (0.035) | 2.760 | erlotinib | PR | 5 | W |
| #092 | W | W (0) | ND | TN | W | W (0.047) | 0 | TN | W (0) | ND | erlotinib | PR | 24+ | W |
| #053 | ND | W (0.093) | Failed | TN | ND | W (0.029) | Failed | TN | W (0.079) | NA | gefitinib | PR | 15 | W |
| #116 | W | Mut (3.172) | 0.500 | TP | Mut | Mut (2.315) | Failed | TP | W (0) | ND | gefitinib | SD | 3 | Mut |
| #076 | Mut | Mut (0.180) | 0.800 | TP | W | Mut (0.162) | 0 | FP | Mut (0.417) | 0.230 | gefitinib | SD | 8 | Mut |
| #004 | Mut | W (0.004) | 0.040 | FN | W | W (0.016) | 0.010 | TN | W (0.037) | 0 | erlotinib | SD | 20 | Mut |
| #006 | W | Mut (0.247) | 0.034 | TP | W | Mut (0.226) | 0 | FP | W (0) | ND | erlotinib | SD | 17 | Mut |
| #047 | W | W (0.012) | 0.029 | TP | W | Mut (2.103) | 9.200 | TP | W (0.041) | 0 | gefitinib | SD | 7 | Mut |
| #021 | W | W (0) | ND | TN | W | W (0.014) | 0.024 | TP | W (0.046) | 0.015 | erlotinib | SD | 3 | Mut |
| #035 | W | W (0) | ND | TN | W | W (0) | ND | TN | W (0.092) | 0 | erlotinib | SD | 2 | W |
| #075 | W | W (0.145) | 0.014 | TP | W | W (0) | ND | TN | W (0.109) | 0.028 | erlotinib | SD | 2 | Mut |
| #095 | W | W (0) | ND | TN | W | W (0) | ND | TN | W (0.119) | Failed | erlotinib | SD | 24+ | W |

ND: Not done; NT: Not treated; NA: Not available; NE: Not evaluable; PR: Partial response; SD: Stable disease; PD: Progressive disease.
